# Gingyo-San Enhances Immunity and Potentiates Infectious Bursal Disease Vaccination

**DOI:** 10.1093/ecam/nep021

**Published:** 2010-10-25

**Authors:** Che-Ming Hung, Chia-Chou Yeh, Kowit-Yu Chong, Hsiao-Ling Chen, Jiun-Yu Chen, Shung-Te Kao, Chih-Ching Yen, Ming-Hsien Yeh, Maw-Sun Lin, Chuan-Mu Chen

**Affiliations:** ^1^Animal Industry Division, Livestock Research Institute, Council of Agriculture, Tainan 712, Taiwan; ^2^Department of Life Sciences, National Chung Hsing University, Taichung 402, Taiwan; ^3^Department of Chinese Medicine, Buddhist Dalin Tzu Chi General Hospital, Chia-Yi 622, Taiwan; ^4^Department of Medical Biotechnology and Laboratory Science, Chang Gung University, Tao-Yuan 333, Taiwan; ^5^Department of Molecular Biotechnology, Da-Yeh University, Changhwa 515, Taiwan; ^6^China Medical University Hospital, Taichung 404, Taiwan

## Abstract

The purpose of the present study was to investigate the effects of Gingyo-san (GGS), a traditional Chinese medical formula, on peripheral lymphocyte proliferation and serum antibody titers in chickens vaccinated against the infectious bursal disease (IBD) virus. Treatment groups were fed one of three doses of GGS in their diet (0.5%, 1.0% and 2.0%, w/w), and the IBD vaccine was administered at 1 and 3 weeks of age. At Weeks 8, 12 and 16, changes in serum IBD antibody titers were measured via the micro-method and T cell proliferation. In gene expression experiments, GGS-treated peripheral T lymphocytes were stimulated with concanavalin A (ConA) for 24 h. The mRNA expression of interleukin-2 (IL-2), interferon-*γ* (IFN-*γ*), interleukin-4 (IL-4) and interleukin-12 (IL-12) was determined using a semi-quantitative RT-PCR assay. The results showed that a low dose of GGS could significantly raise the antibody titers. Medium and high doses of GGS enhanced IL-2 and IFN-*γ* production. GGS altered the expression of IL-4 and IL-12 in T lymphocytes. CD4^+^ T lymphocyte development was also skewed towards the Th1 phenotype. GGS enhanced cell-mediated immunity and augmented the effects of IBD vaccination in strengthening subsequent anti-viral responses.

## 1. Introduction

Many traditional Chinese medicines (TCM) and their ingredients have been reported to enhance immunity [1–3], and they have great potential in many practical applications. Immunomodulation is an important process for infectious diseases, especially viral diseases. These diseases result in huge losses in the domestic animal and poultry industries [4]. Some infectious diseases remain hard to control due to the heterogeneity of microorganisms, the inferior quality or improper storage and transport of vaccines, and the occurrence of immunosuppressive diseases. The use of an immunopotentiator with a vaccine could improve the efficacy and decrease the toxicity of vaccination [5].

Infectious bursal disease (IBD) has a sudden onset with a short incubation period (2-3 days). Morbidity is usually 100%, but mortality varies depending on the virus strain [4, 6, 7]. The IBD virus is ubiquitous, is resistant to a variety of disinfectants, and is environmentally stable. Chickens are widely exposed to the IBD virus worldwide, which leads to multi-billion dollar losses in the poultry industry [8]. Serological evidence of natural infection with the virus showed infection levels of 58.6% in Taiwan [9]. Strategies to control IBD are largely based on vaccination programmes.

In TCM, Gingyo-san (GGS), is a crude drug containing extracts from 10 medicinal plants. In TCM therapy, GGS is frequently used to treat pulmonary disorders including the common cold and bronchial infections [2]. Experiments conducted in Japan have revealed that GGS has antipyretic and antiviral effects [10, 11]. Pharmacological studies of GGS indicate that it is effective at alleviating fever, relieving pain, counteracting hypersusceptibility and counteracting bacterial and viral infections [12]. In animal trials, GGS also exhibited significant therapeutic effects on bacterial and viral infections in mice [13]. In addition, two components identified from GGS were shown to exhibit antiviral activities in mice infected with the influenza virus [11, 14]. Recently, it has been reported that GGS exerts an immunomodulatory effect during acute respiratory distress syndrome (ARDS), through the down-regulation of inflammatory cytokines and the up-regulation of anti-inflammatory cytokines [2]. The use of GGS in feed additives as part of a preventative programme has been implemented on domestic farms in China.

In this study, we investigated the possibility of using GGS as an immune stimulator to enhance peripheral lymphocyte proliferation and serum antibody titers in chickens vaccinated against the IBD virus. The dose-dependent responses of immune enhancement to GGS were also evaluated.

## 2. Methods

### 2.1. Preparation of GGS Extract

**Figure 1:**
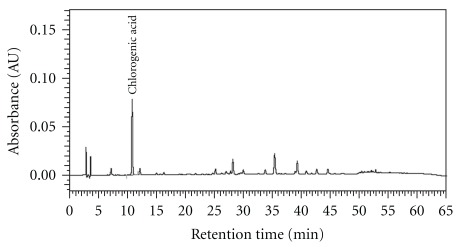
HPLC chromatogram of GGS. The solution of GGS was prepared by dissolving it in pyrogen-free isotonic saline (10 mg/100 mL). The major active compound of chlorogenic acid (0.081 mg mL^−1^) was detected at 10.77 min retention time. The injection volume was 10 *μ*L, and the flow rate was 1.0 mL min^−1^.

Medicinal plants were provided by Koda Pharmaceutics Ltd (Taoyuan, Taiwan) for the preparation of the GGS extract. GGS is commercially available in Taiwan and Japan. The preparation is a mixture of 10 crude plant ingredients: *Lonicera japonica* Thunb. (herb. no. 10802; Caprifoliaceae), *Forsythia suspensa* Thunb. (herb. no. 11005; Oleaceae), *Mentha haplocalyx* Briq. (herb. no. 11604; Lamiaceae), *Schizonepeta tenuifolia* Briq. (herb. no. 11002; Lamiaceae), *Glycine max* Merr. (herb. no. 11105; Fabaceae), *Glycyrrhiza uralensis* Fisch. (herb. no. 10503; Fabaceae), *Platycodon grandiflorum* Jacq. (herb. no. 11004; Campanulaceae), *Lophatherum gracile* Brongn. (herb. no. 11106; Poaceae), *Arctium lappa* L. (herb. no. 10404; Asteraceae) and *Phragmites communis* Trin. (herb. no. 12001; Poaceae), at a ratio of 10 : 10 : 4 : 4 : 5 : 5 : 6 : 5 : 6 : 10. The voucher specimen and method of extraction and analysis of GGS were as described previously [2]. The extract was dissolved in pyrogen-free isotonic saline (YF Chemical, Taipei, Taiwan) and filtered through a 0.2 mm filter (Microgen, Laguna Hills, CA, USA) before use. The high-performance liquid chromatography (HPLC) chromatogram of the GGS for quality control is shown in Figure 1.

### 2.2. Reagents

RPMI-1640 medium (Roswell Park Memorial Institute-1640 medium; GIBCO Invitrogen, Carlsbad, CA), supplemented with benzylpenicillin (100 IU mL^−1^) and streptomycin (100 IU mL^−1^), was used for *invitro* cell culture, washing and re-suspending cells, and diluting the mitogen [15]. Concanavalin A (ConA) (Sigma Chemicals, St Louis, MO) was dissolved in the RPMI-1640 medium to a final concentration of 0.025 mg mL^−1^. 3-(4, 5-Dimethylthiazol-2-yl)-2,5-diphenyl-tetrazolium bromide (MTT, Amersco Inc., Solon, OH) was dissolved in calcium- and magnesium-free (CMF) phosphate-buffered saline (PBS, pH 7.4) to achieve a concentration of 5 mg mL^−1^. These reagents were filtered through a 0.22 *μ*m filter [16, 17]. The ConA solution was stored at −20°C and the MTT solution and RPMI-640 media were stored at 4°C. The lymphocyte separation medium (Ficoll-Hypaque) and dimethyl sulphoxide (DMSO) were purchased from Sigma Chemicals.

### 2.3. Animals

One-day-old Taiwanese broilers were purchased from the Livestock Research Institute and reared in concrete floor pens (1.8 m × 1.8 m per pen) padded with rice hull litter in an open-sided growing house until 16 weeks of age. The chickens were fed the basal corn-soybean meal diets based on the guidelines suggested by National Research Centre (NRC) [18] and the Extension Booklet for the Taiwan country broiler [19]; these guidelines were formulated to meet the nutritional requirements for Taiwan country broilers at different stages of the life cycle. Feed and water were supplied *ad libitum*. All experimental procedures were approved by the Institutional Animal Care and Use Committee of the Livestock Research Institute.

### 2.4. Experimental Design

Forty chickens were randomly divided into four groups containing equal numbers of birds of similar body weights (the average BW was 32 g). Group 1 was the medicine-free control. Groups 2–4 were fed GGS powder at one of three concentrations: 0.5% (w/w, low dosage), 1% (medium dosage) and 2% (high dosage), respectively. The IBD vaccine was administered twice at 14-day intervals. The first vaccine (Bursine-2, Fort Dodge Animal Health Inc., Charles, IA) was orally administered at 1 week of age and the second (IBD BLEN, Merial Select Inc., Gainesville, GA) at 3 weeks of age. At Weeks 8, 12 and 16, six chickens were sampled randomly from each group for the examination of peripheral lymphocyte proliferation by the MTT assay. At Weeks 8 and 16, eight additional chickens were sampled randomly from each group for the determination of serum IBD antibody titers using an enzyme-linked immunosorbent assay (ELISA) kit [20]. The animal trials were repeated twice.

### 2.5. Blood Sample Collection and Analysis of Biological Parameters

Blood samples were collected from the wing vein with a 24G needle. The tubes were allowed to stand for 30 min and then centrifuged at 800 × g for 15 min to isolate the serum. Thereafter, the sera were stored at –20°C until analysis. The concentrations of glutamic oxaloacetic transaminase (GOT) and glutamic pyruvic transaminase (GTP) were determined by the standardized method of the Japan Society of Clinical Chemistry (JSCC method kit) (Wako Pure Chem., Osaka, Japan). Blood urea nitrogen (BUN) contents were determined by a Urase-glutamate dehydrogenase (Urease-GLDH) kit (Wako). Creatinine (CRE) levels were determined using a Jaffe kit (Wako). Triglycerides (TG) were assessed using a free glycerol blanking kit (Wako). Total cholesterol (T-Chol) was determined with a Cholesterol Oxidase-HDAOS kit (Wako).

### 2.6. Jejunum Villous Measurement

Intestinal samples were taken on the last day of the experiment. The intestinal sections (2.5 cm) from six chickens per treatment group were obtained from the jejunum at a position midway between the Meckel's diverticulum and the entrance of the bile ducts. The samples were flushed with saline, fixed in 10% buffered formalin (pH 7.0), embedded with paraffin, sectioned into 3 *μ*m sections and stained with haematoxylin-eosin (IDEXX Veterinary Services, Sacramento, CA). For each intestinal section, 10 randomly selected jejunum villi heights were estimated using Image-Pro-Plus software (Media Cybernetics, Silver Spring, MD).

### 2.7. Peripheral Lymphocyte Proliferation Assay

To detect changes in cellular immunity, a peripheral lymphocyte proliferation assay was performed, as described previously [5]. Blood samples (5 mL per chicken) were collected and immediately transferred into aseptic capped tubes containing sodium heparin. The samples were diluted with an equal volume of Hanks' solution and carefully layered on the surface of the lymphocyte separation medium. After 15 min centrifugation at 800 × g, a white cloud-like band was observed in the lymphocyte separation solution interface. The lymphocyte band was collected and washed twice with RPMI-1640 media without fetal bovine serum (FBS). After centrifugation, the pellet was resuspended in RPMI-1640 media at a concentration of 5 × 10^6^ cells per mL, and 80 *μ*L per well was incubated in 96-well tissue culture plates. Another 20 *μ*L of ConA was added to each well, and each sample was seeded in four wells. A working concentration of 5 *μ*g mL^−1^ ConA was used for the stimulation of lymphocytes. After 44 h of incubation at 39.5°C in the 5% CO_2_ incubator, 20 *μ*L of MTT (5 mg mL^−1^) was added to each well. The plates were incubated for another 4 h, and then 100 *μ*L of DMSO was added to each well; the plates were finally shaken for 5 min to completely dissolve the precipitate. Light absorbance at 570 nm was measured with an ELISA plate reader (Bio-Rad Laboratories Inc., Irvine, CA).

### 2.8. Measurement of IgA, IgG and IBD Antibodies in Serum

Total IgA and IgG concentrations in the sera were measured with the Chicken IgA and IgG ELISA Quantitation Kit (Bethyl Laboratories, Inc., Montgomery, TX) according to the manufacturer's instructions. The sera were diluted up to 500-fold with a sample diluent solution from the kit, and read at 450 nm in an ELISA plate reader [21]. The antibody titer of IBDV was determined using ELISA kits (Kirkegaard & Perry Laboratories Inc., Gaithersburg, MD), according to the manufacturer's instructions. Twenty microliters of buffer serum from each chicken was used and read at 650 nm in an ELISA plate reader [21].

### 2.9. Detection of Cytokine mRNA by Semi-Quantitative RT-PCR

The mRNA expression of the cytokines IL-2, INF-*γ*, IL-4 and IL-12 was determined with a reverse transcriptional polymerase chain reaction (RT-PCR). The housekeeping gene, GAPDH, was used as an internal control. Peripheral lymphocytes were collected from the GGS-treated and untreated groups and harvested at 24 h after ConA stimulation. Total RNA was isolated from the tissues using the TRIzol reagent (Invitrogen Life Technologies, Carlsbad, CA), according to the manufacturer's instructions. The RNA was subsequently treated with Deoxyribonuclease I (MBI Fermentas Inc., Lithuania) to remove any genomic DNA contamination [22]. Approximately 900 ng of total RNA was reverse-transcribed with MuLV reverse transcriptase using the GeneAmp RNA PCR Kit (Applied Biosystems, Foster, CA) and oligo d(T)16 primers. The RT-PCR primer sequences and annealing conditions for IL-2, IL-4, IL-12, INF-*γ* and GAPDH are listed in Table 1. The amplified RT-PCR products were subjected to electrophoresis at 100 V in a 2% agarose gel (Invitrogen, Carlsbad, CA) for about 30 min [23]. The agarose gels were stained with 0.5 mg mL^−1^ ethidium bromide Tris/borate/EDTA buffer (ICN, Costa Mesa, CA).

### 2.10. Statistical Analysis

The experimental data were analysed using the General Linear Model (GLM) procedure by SAS, as described previously [24]. The differences among herbal medicinal dosage and control groups were compared by least mean squares comparison. Statistical significances were based on *P < .05 and **P < .01.

## 3. Results

### 3.1. Assessment of Biochemical Functions in Serum

We tested the effects of the herbal remedies on serum lipids, liver and kidney function. Feeding the chickens a diet supplemented with GGS for 16 weeks had no effect on body weight or composition (data not shown). The bioactivity indices for liver and kidney functions in the sera were measured before and after the experiment. The GOT and GPT were not significantly different between the control and tested groups; however, the BUN concentrations were significantly decreased in all GGS-treated groups (*P* <  .05), and the CRE concentration was also decreased in the middle-dose GGS-treated group (*P* <  .05), indicating that the dietary supplements of GGS modulated kidney function. The GGS containing the active component of chlorogenic acid (Figure 1) significantly reduced serum cholesterol in all three GGS-treated groups (*P* <  .05), but had no effect on the triglycerides (Table 2).

### 3.2. Physical Characteristics of the Jejunum

**Figure 2:**
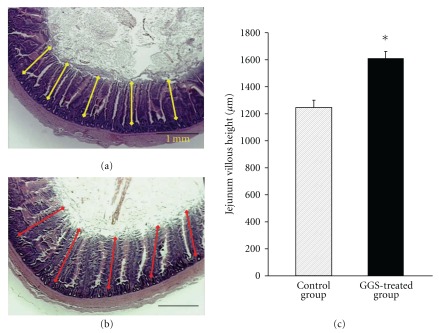
Effect of GGS on jejunum. Jejunum histology and villous length of chickens fed a basal diet without GGS (control) (a) compared to those fed a basal diet with 0.5% GGS (treatment) (b). For each intestinal section, the jejunum villous height was estimated for at least 10 individual villi in the control and GGS-treated groups, as shown by the yellow and red lines, respectively. The scale bar represents 1 mm. (c) Mean ± SE of jejunum villous height (*μ*m) in control and treated groups. *P <  .05.

Physical changes were seen in the intestines of the birds that were given the antibiotic growth promoter and health factor. The jejunum histology and villous length of chickens fed a diet with or without GGS at 16 weeks are shown in Figure 2. The villous length was significantly higher in the GGS-treated group than in the control group (1250 ± 53 versus 1603 ± 72 *μ*m; *P* <  .05), indicating that dietary supplementation with GGS is beneficial to the health of the chicken.

### 3.3. Peripheral Lymphocyte Proliferation in Response to GGS

To investigate the effects of GGS on peripheral lymphocyte proliferation, we collected peripheral lymphocytes from chickens treated with different dosages of orally administered GGS, and treated them with or without ConA *invitro*. Proliferation was measured via the MTT assay, and changes in the proliferation ratio (ConA+/ConA–) are summarized in Table 3. At Weeks 8, 12 and 16, peripheral lymphocyte proliferation in the GGS_M_-treated group was slightly increased; however, proliferation in the GGS_H_-treated group was significantly higher than in the control group, by 2-3-fold (*P* < .05).

### 3.4. Systemic Antibody Response

**Figure 3:**
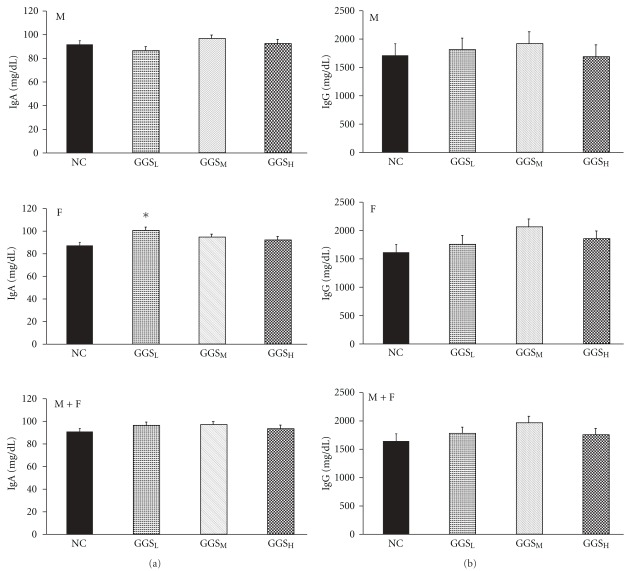
Effect of GGS on serum IgA (a) and IgG (b). The negative control (NC) group was fed with a basal diet without GGS. The levels of serum IgA and IgG (mg dL^−1^) in the 0.5, 1 and 2% GGS-treated native chicken groups are shown as GGS_L_, GGS_M_ and GGS_H_, respectively. Blood samples were collected at 16 weeks of age. Upper panel (M): Data from male chickens in each group. Middle panel (F): Data from female chickens in each group. Lower panel (M + F): Combined male and female data. The asterisks represent significant differences (*P* <  .05) compared with the respective control groups.

To evaluate the effects of GGS on the serum antibodies, the presence of natural antibodies to IgA (Figure 3(a)) and IgG (Figure 3(b)) in the sera of GGS-treated and untreated birds was assessed. In the GGS_L_-treated group only, the sera contained significantly higher levels of IgA antibodies in the females than in the control group (*P* <  .05) (Figure 3(a)). However, there was no significant difference in IgG antibodies between the groups (Figure 3(b)).

### 3.5. Enhanced IBD Antibody Titer in Response to GGS

Changes in the IBD antibody titers in response to GGS are listed in Table 4. In general, the antibody titers of all treatment groups at each time point were higher than those of the control group, and the serum IBD antibody titers in all groups at Week 16 were lower than those at Week 8. After vaccination, the serum antibody titers in the low-dosage GGS_L_-treated groups were significantly higher than those of the control group (*P* <  .05) and other treatment groups at Week 8. At Week 16, the IBD antibody titers of GGS_L_ and GGSM groups were significantly higher than those of the control group (*P* <  .05).

### 3.6. Cytokine Gene Expression

**Figure 4:**
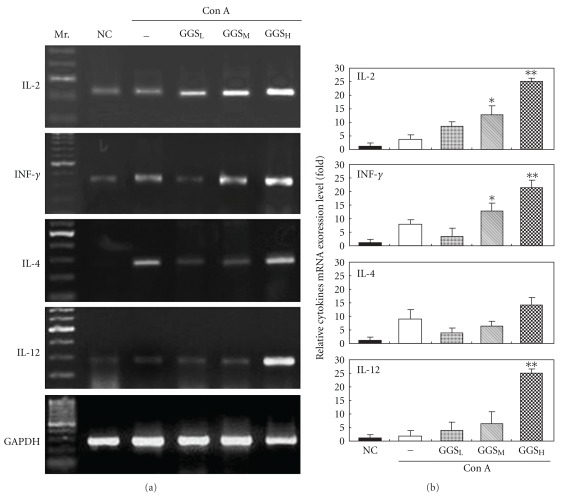
Quantitative analysis of IL-2, INF-, IL-4 and IL-12 mRNA expression in cultured chicken peripheral lymphocytes treated with different GGS doses at 24 h after ConA stimulation. (a) Representation of mRNA expression by semi-quantitative RT-PCR analysis. Mr.: 100-bp ladder of DNA markers; NC: normal cultured chicken peripheral lymphocytes (Week 8) without ConA stimulation; –: no GGS; GGS_L_: low-dose GGS treatment group; GGS_M_: medium-dose GGS treatment group; GGS_H_: high-dose GGS treatment group. The results are representative of three experiments. (b) The relative mRNA expression levels in control and different GGS-treated groups were quantified by a densitometer. The signal from GAPDH RT-PCR products in each sample was used as an internal control in each mRNA calculation. *P <  .05; **P <  .01.

Changes in cytokine expression at Week 8 are shown in Figure 4. In the IL-2 and INF-*γ* networks, the IL-2 and INF-*γ* mRNA levels of the normal control group were low; ConA increased their expression. GGS increased IL-2 expression in the medium-dose groups (*P* <  .05), but significantly increased IL-2 in the high-dose group (*P* <  .01). GGS increased INF-*γ* expression in a dose-dependent manner in all groups except the low-dose group, which demonstrated suppressed INF-*γ* expression. In the IL-4 and IL-12 network, the expression of IL-4 and IL-12 was low in the normal control group. ConA treatment increased the level of IL-4 but not IL-12. We also observed that IL-4 was down-regulated in the low- and medium-dose groups, and slightly increased in the high-dose GGSH-treated group. IL-12 expression was significantly increased in the high-dose GGS-treated group (*P* <  .01).

## 4. Discussion

GGS enhanced the IBD antibody titer at a low dose, and promoted peripheral lymphocyte proliferation at a high dose. GGS regulated the expression of IL-2, INF-*γ*, IL-4 and IL-12 in the ConA-stimulated peripheral T lymphocytes.

Physical changes were observed in the intestines of birds that were given the antibiotic growth promoter and health factor. The growth promoter is known to inhibit normal early microbial proliferation and, hence, the competition for essential nutrients during the gut maturation process in chickens [25]. In our study, GGS increased the length of the jejunum (Figure 2), indicating that GGS could improve anti-microbial proliferation and increase nutrition absorption. GGS was also found to protect kidney function and to decrease cholesterol in GGS-treated groups for a long period of time (Table 2). All of these data suggest that GGS could improve the health of an organism.

A traditional control strategy for IBD in broilers has involved protection via passive immunization [4]. However, traditional inactivated or attenuated vaccines do not provide enough protection against these antigenic variants, which are very virulent (vv) IBDV [26]. Improving the effectiveness of the IBD vaccine is, therefore, an important issue. According to our data, the low dosage GGS-treated group showed enhanced IBD antibody titers at Week 8. The low and medium dosage groups also had significantly higher IBD antibody titers than the control group at Week 16 (Table 4). In a previous report, 1-day-old chickens were fed a diet with or without ascorbic acid and vaccinated against IBD at 7 days of age, and both groups were then challenged orally with 4 × 10^5^ IBDV viral particles at 21 days of age. At 21 days of age (14 days post-vaccination) and 31 days of age (10 days post-challenge), the number of anti-IBDV IgG antibody secreting cells were significantly higher in the ascorbic acid supplemented group than in the control group [27]. A similar study also revealed that orally administered 2% l-arginine elicited a higher antibody titer after IBDV vaccination, and increased protection rate after IBDV challenge, when compared with the control group [28]. These results suggested that increasing the anti-IBDV antibody titer response to IBDV vaccination would increase the protection efficiency against IBDV infection. Most pathogens invade the body of their host through mucosal surfaces, especially those lining the gastrointestinal, respiratory and genito-urinary tracts. Despite their phylogenetic distance, both mammals and bird species use polymeric IgA (pIgA), which is produced by plasma cells in the lamina propria underlying the mucosal epithelia [29], as the primary immunological defense against such infections. It is interesting to note that in our study, a low dosage of GGS increased the level of IgA in the serum of female chickens (Figure 3), indicating that a low dosage of GGS could increase the primary immunological defense in mucosal epithelia. We do not yet understand why this phenomenon only appeared in the females.

IBD causes bursal follicular lymphoid depletion, leading to significant reduction in humoural immune responses. IBDV infection apparently affects immature or precursor B cells to a greater extent than mature B-lymphocytes. The disease is a major problem in concentrated poultry production areas throughout the world. However, it is often not recognized because of its often subclinical presentation. Affected chickens have reduced antibody response to vaccinations, strong postvaccination reactions and increased susceptibility to concurrent or secondary infections [27]. Traditional IBD vaccines have focused on stimulating the B cells. However, recent studies that have focused on the importance of T cells in IBDV pathogenesis, have shown that cell-mediated immunity may be more important in IBDV infections than previously thought [30, 31]. Our data show that a high dose of GGS can improve peripheral T lymphocyte proliferation in response to ConA stimulation and can increase the proliferation rate of these cells in a time-dependent manner beginning at Week 8 (Table 3).

**Figure 5:**
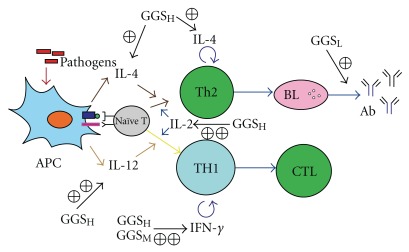
Proposed mechanism of GGS functions in immunity enhancement. High-dose of GGS (GGS_H_) administration will increase IL-2, IL-4 and IL-12 expression from antigen presenting cells (APC) to stimulate naïve T cells differentiation. Administration of a medium dose of GGS (GGS_M_) as well as GGS_H_ will enhance IFN-*γ* expression to stimulate T-helper 1 (Th1) cells differentiation and cytotoxic T lymphocytes (CTL) function. Administration of a low-dose of (GGS_L_) will further increase B lymphocytes (BL) producing specific antibodies.

Interleukin-2 (IL-2), initially known as a T-cell growth factor, is a powerful immuno-regulatory lymphokine that is produced by lectin- or antigen-activated T cells [8, 32, 33]. It is secreted by mature T lymphocytes upon stimulation, and is constitutively secreted by certain T-cell lymphoma cell lines. Chicken IL-2 (ChIL-2) may enhance immunity against avian pathogens, which would introduce a new weapon for the control of infectious diseases in poultry. The administration of the IBD vaccine with ChIL-2 provided better protection than vaccination alone [8]. Previous reports showed that the simultaneous injection of chicken IL-2 plasmid DNA with the IBD vaccine significantly increased protection after challenge with the virulent strain [32, 33]. These results strongly indicate that the efficacy of the avian DNA vaccine could be modulated by the co-administration of a plasmid encoding ChIL-2. In this study, we further demonstrated that a high-dose of GGS strongly enhanced ConA-induced ChIL-2. Therefore, GGS could improve the efficiency of the IBD vaccine. Furthermore, immune cells and immune molecules can mutually regulate one another and form many immune regulation networks. The IL-2-IFN-*γ*-NKC system is one such immune regulation network. In this network, IL-2 plays an important role in regulating the generation of IFN-*γ* and the activity of natural killer cells (NKC). IFN-*γ* also participates in the immune response mediated by T cells, B cells and other immune cells [34]. IFN-*γ* severely restricts virus replication and can even cure persistently infected bovine epithelial cells [35]. Our study showed that the medium- and high-dose GGS-treated groups not only exhibited increased expression of IL-2 mRNA, but also increased expression of IFN-*γ* mRNA (Figure 4). Chlorogenic acid is the most abundant compound in the GGS. In the previous study, it was found that chlorogenic acid could enhance the activity of human lymphocyte proliferation and IFN-*γ* secretion [36]. Thus, it is proposed that GGS strengthened immunity via the mechanism outlined in Figure 5.

In mammals, it has been known for some time that the balance between the Th1 and Th2 lymphocyte subsets determines susceptibility to some disease states. Thus, an unusually dominant Th1 response is often associated with autoimmunity, while improper development of Th2 immunity can lead to allergic diseases [37]. Chicken Th2 cells are necessary to induce the humoural response to combat parasite invasion [38]. As in mammals, the chicken genome contains a cluster of Th2 cytokine genes containing IL-4 and other cytokines that are expressed in lymphoid tissues. Activation of this gene cluster is associated with down-regulating the inflammatory (Th1) response and, as a result, drives Th2 cell development [38]. Unlike IL-4, IL-12 is able to facilitate the proliferation and activation of Thl cells and the production of IL-2 and IFN-*γ* [39]. IL-12 induces IFN-*γ* synthesis by neonatal CD4 T-cells. Additionally, IL-2 can further synergise with IL-12 to trigger IFN-*γ* production [40, 41]. Our data demonstrate that GGS-treated chickens produced more IL-12 than IL-4, and that IL-2 and INF-*γ* were also up-regulated. The results reveal that GGS can skew the lymphocyte subset development towards Th1 rather than Th2 cells.

In conclusion, our results show that GGS-enhanced cell-mediated immunity and assisted the IBD vaccine, strengthening the anti-viral functions. Since the chicken's immune system is similar to that of mammals, chickens provide an attractive model system to study the effectiveness of GGS supplements in controlling diseases in livestock and human beings. Nevertheless, additional investigations are required to determine the potential clinical utility of GGS as an immunopotentiator in infant vaccination programmes. The pharmaceutical technology of TCM as applied in animal health care is relatively simple, and its production costs are relatively low.

## Funding

Council of Agriculture [95AS-5.1.2-LI-L1(3), partial], National Science Council, and the Ministry of Education, Taiwan, Republic of China, under the ATU plan (NSC-97-2313-B-005-004-MY3).

## Figures and Tables

**Table 1:** Oligonucleotide primer sets used for semi-quantitative RT-PCR.

RNA target	Primer sets	Oligonucleotide sequence	Annealing temperature (°C)	Product size (bp)	Accession no.
INF-*γ*	F	5′-AGCTGACGGTGGACCTATTA-3′	56	259	NM_205149
	R	5′-GGCTTTGCGCTGGATTCTC-3′			
IL-2	F	5′-CTGGGACCACTGTATGCTCT-3′	55	256	AF017645
	R	5′-CACCAGTGGGAAACAGTATC-3′			
IL-4	F	5′-ACCCAGGGCATCCAGAAG-3′	55	258	AJ621735
	R	5′-CAGTGCCGGCAAGAAGTT-3′			
IL-12	F	5′-AGACTCCAATGGGCAAATGA-3′	56	244	NM_213571
	R	5′-CTCTTCGGCAAATGGACAGT-3′			
GAPDH	F	5′-GGTGGTGCTAAGCGTGTTAT-3′	56	264	
	R	5′-ACCTCTGTCATCTCTCCACA-3′			

**Table 2:** The effects of different supplemental dosages of GGS on serum parameters in chickens.

Item	Control group	Treated group^a^			SE
		GGS_L_	GGS_M_	GGS_H_	
GGS Dose (%, w/w)	0	0.5%	1%	2%	
GOT (UL^−1^)	195.3	171.3	183.8	182.5	13.4
GPT (UL^−1^)	7.7	6.8	7.7	6.8	0.8
Cholesterol (md dL^−1^)	109.3	90.8*	83.8*	80.7*	5.9
Triglycerides (md dL^−1^)	27.8	33.5	21.3	24.8	6.9
BUN (md dL^−1^)	2.4	1.9*	1.6*	1.6*	0.2
Creatinine (md dL^−1^)	0.28	0.23	0.20*	0.25	0.02

^a^GGS_L_: low dose of GGS; GGS_M_: medium dose of GGS; GGS_H_: high dose of GGS.

*Significant difference (*P* <  .05 versus control group).

**Table 3:** The effects of supplemental GGS on peripheral lymphocyte proliferation in chickens of different ages.

Group^a^	Dose (%, w/w)	Lymphocyte proliferation ratio		
		(ConA+/ConA–)		
		8 weeks	12 weeks	16 weeks
Control	0	1.90	2.13	1.87
GGS_L_	0.5	1.77	2.18	1.72
GGS_M_	1.0	2.40	2.68	2.79
GGS_H_	2.0	3.09*	3.76*	5.24*
SE		0.34	0.31	0.52

^a^GGS_L_: Low dose of GGS; GGS_M_: medium dose of GGS; GGS_H_: high dose of GGS.

*Significant difference (*P* <  .05 versus control group).

**Table 4:** The effects of supplemental GGS on the IBD antibody titer (×10^3^) in chickens of different ages.

Group^a^	Dose (%, w/w)	IBD antibody titer (×10^3^)	
		8 weeks	16 weeks
Control	0	5.892	5.080
GGS_L_	0.5	11.006*	8.191*
GGS_M_	1.0	9.935	9.819*
GGS_H_	2.0	6.831	6.640
SE		1.461	0.968

^a^GGS_L_: low dose of GGS; GGS_M_: medium dose of GGS; GGS_H_: high dose of GGS.

*Significant difference (*P* <  .05 versus control group).
